# CCL-2 and CXCL-8: Potential Prognostic Biomarkers of Acute Kidney Injury after a *Bothrops atrox* Snakebite

**DOI:** 10.1155/2022/8285084

**Published:** 2022-09-07

**Authors:** Juliana Costa Ferreira Neves, Hiochelson Najibe Santos Ibiapina, Fábio Magalhães-Gama, Jacqueline Almeida Gonçalves Sachett, Iran Mendonça Silva, Kerolaine Fonseca Coelho, Eliane Campos Alves, Andréa Monteiro Tarragô, Luiz Carlos de Lima Ferreira, Adriana Malheiro, Wuelton Marcelo Monteiro, Allyson Guimarães Costa

**Affiliations:** ^1^Post-graduate Program in Tropical Medicine, Amazonas State University (UEA), Manaus, AM, Brazil; ^2^Carlos Borborema Clinical Research Institute, Tropical Medicine Foundation Doctor Heitor Vieira Dourado (FMT-HVD), Manaus, AM, Brazil; ^3^Post-Graduate Program in Basic and Applied Immunology, Federal University of Amazonas (UFAM), Manaus, AM, Brazil; ^4^Post-Graduate Program in Health Sciences, René Rachou Institute, Oswaldo Cruz Foundation (FIOCRUZ-Minas), Minas Gerais, Belo Horizonte, Brazil; ^5^Directorate of Teaching and Research, Hematology and Hemotherapy Foundation of Amazonas (HEMOAM), Manaus, AM, Brazil; ^6^Department of Education and Research, Alfredo da Matta Foundation (FUAM), Manaus, AM, Brazil; ^7^Post-Graduate Program in Sciences Applied to Hematology, UEA, Manaus, AM, Brazil; ^8^Nursing School of Manaus, UFAM, Manaus, AM, Brazil

## Abstract

In the Brazilian Amazon, the snake *Bothrops atrox* is the primary cause of snakebites. *B. atrox* (BaV) venom can cause systemic pathophysiological changes such as acute kidney injury (AKI), which leads to the production of chemokines and cytokines in response to the envenomation. These soluble immunological molecules act by modulating the inflammatory response; however, the mechanisms associated with the development of AKI are still poorly understood. Here, we characterize the profile of these soluble immunological molecules as possible predictive biomarkers of the development of AKI. The study involved 34 patients who had been victims of snakebites by *Bothrops* sp. These were categorized into two groups according to the development of AKI (AKI^(-)^/AKI^(+)^), using healthy donors as the control (HD). Peripheral blood samples were collected at three-time points: before antivenom administration (T0) and at 24 and 48 hours after antivenom (T1 and T2, respectively). The soluble immunological molecules (CXCL-8, CCL-5, CXCL-9, CCL-2, CXCL-10, IL-6, TNF, IL-2, IL-10, IFN-*γ*, IL-4, and IL-17A) were quantified using cytometric bead array. Our results demonstrated an increase in CXCL-9, CXCL-10, IL-6, IL-2, IL-10, and IL-17A molecules in the groups of patients who suffered *Bothrops* snakebites (AKI^(-)^ and AKI^(+)^) before antivenom administration, when compared to HD. In the AKI^(+)^ group, levels of CXCL-8 and CCL-2 molecules were elevated on admission and progressively decreased during the clinical evolution of patients after antivenom administration. In addition, in the signature analysis, these were produced exclusively by the group AKI^(+)^ at T0. Thus, these chemokines may be related to the initiation and extension of AKI after envenomation by *Bothrops* and present themselves as two potential biomarkers of AKI at T0.

## 1. Introduction

Snakebites represent a neglected public health problem with high morbidity and mortality rates in tropical and subtropical countries [1, 2]. In Brazil, the *Bothrops* genus is widely distributed, with approximately 29 species throughout the national territory, and in the Brazilian Amazon, *Bothrops atrox* is responsible for 90% of all cases of venomous snakebite [3–5].

*B. atrox* venom (BaV) has a high proinflammatory capacity that induces leukocyte recruitment and the release of inflammatory mediators, which increase vascular permeability and cause the formation of edema [6–9]. An increase in circulating levels of inflammatory molecules such as chemokines and cytokines is also observed in *Bothrops* envenomations [10, 11]. The exacerbated immune mechanisms in response to envenomation may play an important role in the emergence of local and systemic complications, such as acute kidney injury (AKI), in which the deposition of immune complexes in glomeruli and vessels has been observed in snakebites [12].

As a result of the bite of viperids, AKI is a common complication, with an incidence that varies between 1.4 and 38.5%. It is the main cause of death and may manifest itself in the first hours after the bite [10–15]. Kidneys become vulnerable to damage by the various toxins found in BaV due to the high vascularization of the organ [16]. Metalloproteases (MPs), serinoproteases (SPs), phospholipases A_2_ (PLA_2_), and L-amino acid oxidases (LAAO) are the main enzymes present in BaV and are responsible for clinical and renal manifestations after envenomation [17–20].

The pathogenesis of AKI remains poorly described, but factors such as bleeding, inflammatory processes, fibrin deposition in the renal tubules, immune complexes formation, and the venom's direct action may be related [18, 21–23]. Studies with different *Bothrops* species have shown that BaV can lead to renal damage and proximal tubular damage after the bite [22, 24]. In addition, cytokines can be released by leukocytes and renal tubular cells after cell injury and are important components of the initiation and extension of inflammation in AKI [25]. Proinflammatory molecules, such as CCL-2, CXCL-2, and IL-6, have been found to be elevated in the kidneys after AKI due to an ischemic process [26–28].

In the last years, several new urinary biomarkers have been presented as markers of kidney injury [29]. The molecules CXCL-8 and CCL-2 have been described as participating molecules in kidney disorders of different orders, such as lupus nephritis, where the urinary excretion of CXCL8 is related to renal inflammatory activity [30]. Other studies corroborate the action of CCL-2 on the proteinuria of patients with glomerular alterations [31]. In addition, Stangou et al. suggest CCL-2 as a predictor molecule of renal function in nephropathy [32].

Furthermore, soluble immunological molecules have been correlated with the development of AKI after envenomation by *Bothrops* and may play a role as predictive markers of this complication. Thus, the identification of immunological biomarkers can help reduce these patients' hospitalization period, which can reduce costs for health services since the components currently analyzed (i.e., creatinine) detect AKI late and reduce therapeutic interventions [33]. The data presented here demonstrate that immunological molecules can be potential predictive biomarkers of AKI in bites by *B. atrox* and evidence the participation of the inflammatory process in the pathogenesis of this complication.

## 2. Materials and Methods

### 2.1. Ethical Aspects

This study was approved by the Research Ethics Committee of the Fundação de Medicina Tropical Dr. Heitor Vieira Dourado (FMT-HVD) (process #492,892). Participants read and signed the informed consent form (ICF). The procedures performed are in accordance with the Declaration of Helsinki and Resolution 466/12 of the National Health Council for research involving human beings. Furthermore, this study was submitted to Brazilian Registry of Clinical Trials (ReBEC) (UTN code: U1111-1169-1005).

### 2.2. Study Design

An observational, longitudinal, and prospective study was carried out at FMT-HVD with patients who suffered snakebites caused by a snake of the genus *Bothrops* and sought medical assistance. The measurement of molecules and formation of the control group was performed in partnership with Fundação Hospitalar de Hematologia e Hemoterapia do Amazonas (HEMOAM) in the period between July 2014 and July 2016.

### 2.3. Patients and Sampling

**Figure 1:**
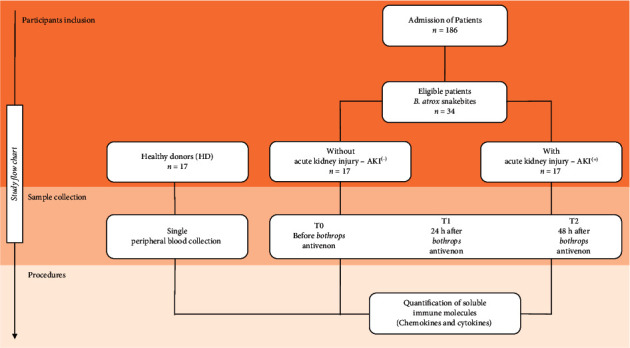
Flowchart of study. Thirty-four patients were considered eligible and were divided into 2 groups according to clinical evolution: without acute kidney injury (AKI^(-)^) and with acute kidney injury (AKI^(+)^). The immunological molecules were quantified using the CBA (cytometric bead array) technique before antivenom administration (T0) and after antivenom administration (T1-24 h and T2-48 h).

The study population consisted of 186 individuals who suffered snakebites caused by a snake of the genus *Bothrops* and sought medical care at the FMT-HVD. Of these, 34 were eligible for the study after evaluation for AKI development [34]. The patients included were divided into two subgroups, referred to as follows: without acute kidney injury (AKI^(-)^), which was composed of patients who suffered envenomation that was classified as mild and did not develop severe systemic alterations; and with acute renal failure (AKI^(+)^), which included patients who suffered severe envenomation and developed AKI during the hospital stay (Figure 1). All patients were classified and treated according to the guideline recommended by the Brazilian Ministry of Health, receiving the Antibotropic serum (SAB), produced at Instituto Butantan, ranging from 2 to 4 ampoules for patients classified as mild, and 12 ampoules for patients classified as severe. This study also included 17 healthy blood donors of either sex with no history of snakebite to constitute the control group (HD). Pregnant victims, indigenous populations, individuals under the age of 18, or those who reported having a history of previous inflammatory diseases, such as autoimmune diseases or immunodeficiency, were not included in the study.

### 2.4. Classification and Definition of Acute Kidney Injury

The classification of patients was performed according to the guidelines of the Brazilian Ministry of Health, which stratifies envenomations into mild, moderate, and severe, based on the local and/or systemic manifestations presented [1]. According to the guidelines provided by the Acute Kidney Injury Network (AKIN), AKI was defined and took into account serum creatinine and diuresis levels [35].

### 2.5. Collection of Biological Samples and Obtaining of Clinical and Laboratory Data

Patients were monitored at three moments: before antivenom administration (T0), at 24, and at 48 hours (T1 and T2, respectively) after antivenom administration. An aliquot of 4 mL of peripheral blood was collected by venipuncture and stored in EDTA tubes (BD Vacutainer® EDTA). After processing, plasma samples were stored in a freezer at -80°C to detect the presence of envenomation and subsequent dosage of chemokines and cytokines. Participants answered a questionnaire with sociodemographic and epidemiological variables to obtain clinical and epidemiological data, and clinical information present in electronic medical records (*iDoctor*) of the FMT-HVD was also used.

### 2.6. Diagnosis of Bothrops Envenomation Using the Enzyme-Linked Immunosorbent Assay (ELISA) Technique

Confirmation of envenomation by snakes of the *Bothrops* genus was performed using the ELISA (enzyme-linked immunosorbent assay) technique at Instituto Butantan as described in a previous study [36]. Biotin-conjugated antivenom detection antibodies were used to identify the venom in peripheral blood.

### 2.7. Quantification of Immunological Molecules

The soluble molecules CXCL-8, CCL-5, CXCL-9, CCL-2, CXCL-10, IL-6, TNF, IL-2, IL-10, IFN-*γ*, IL-4, and IL-17A were quantified using the cytometric bead array (CBA) technique. The BD™ Chemokines Kit (Code No. 552990, BD® Biosciences, San Diego, CA, USA) and BD™ Cytokines Th1, Th2, and Th17 Kit (Code 560484, BD® Biosciences, San Diego, CA, USA) were used following the guidelines described by the manufacturer. The FACS Canto II flow cytometer (BD® Biosciences, San Jose, CA, USA) at HEMOAM was used for sample acquisition. FCAP-Array™ software (v3.01) was used to calculate chemokine and cytokine concentrations in pg/mL and MFI.

### 2.8. Statistical Analysis

Statistical analyses were performed using GraphPad Prism (v8.0.1) and Stata (v13.0) software. The Shapiro-Wilk test was performed for each variable to verify data normality and revealed a nonparametric distribution. The comparison of the values between the two groups was performed using the Mann–Whitney test, while for the comparison of variables with three or more groups, the Kruskal-Wallis test was applied, followed by Dunn's posttest for multiple comparisons between groups. Statistical significance was considered in all cases at *p* < 0.05.

### 2.9. Signature of Immunological Molecules

Immunological molecules or biomarker signature analysis was carried out as described previously [37, 38], by converting the original results of each variable expressed as a continuous variable into categorical data. The cut-off point to converter data were chosen based on the global median values of each soluble molecules, considering the data set that included all groups (HD, AKI^(-)^, and AKI^(+)^). These values were employed to classify the patients for each group as they present “High” (above the cut-off) or “Low” (below the cut-off) of a given soluble molecule. The following cut-offs were used: CXCL‐8 = 506.22; CCL‐5 = 224950.44; CXCL‐9 = 2342.34; CCL‐2 = 1081.08; CXCL‐10 = 2090.66; IL‐6 = 257.40; TNF = 97.24; IL‐2 = 155.87; IL‐10 = 141.57; IFN = 94.38; IL‐4 = 165.88; and IL‐17A = 102.96 expressed in MFI. The Ascendant Biomarker Signatures were assembled in column charts (Symbols & lines) using the 50th percentile as a threshold to identify molecules with increased levels in a higher proportion of individuals. Soluble molecules were underscored and considered relevant when the frequency of individuals with high levels reached values above 50%. In addition, Venn diagram was performed to identify common and selective attributes among each clinical group, using the website http://bioinformatics.psb.ugent.be/webtools/Venn/.

### 2.10. Immunological Molecule Networks

To create the networks, Spearman's correlation test was applied. Positive and negative correlations were considered significant when *p* < 0.05. Then, the Cytoscape 3.0.3 software (Cytoscape Consortium San Diego, CA, USA) was used following the manufacturer's recommendations and instructions. The correlation index (*r*) was used to categorize the strength of correlation as weak (*r* ≤ 0.35), moderate (*r* ≥ 0.36 to *r* ≤ 0.67), or strong (*r* ≥ 0.68), as described above [39, 40].

## 3. Results

### 3.1. Characterization of the Study Population and Laboratory Data

Table 1 summarizes the clinical and epidemiological characteristics of the patients. Males were the most often bitten, with a similar mean age between groups. Most accidents occurred in rural areas, and the most affected anatomical region was the foot in both groups. AKI^(-)^ patients were classified as mild, while the group AKI^(+)^ was classified as severe. Most participants from both groups arrived at the service within 3 hours after the accident. In the AKI^(+)^ group, 59% of patients developed this complication before 24 h of hospitalization.

Table 2 shows the main laboratory parameters evaluated in patients who suffered *Bothrops* snakebites. The analysis was performed with the values presented at their arrival at the emergency service. The values of lactate dehydrogenase (LDH) and creatinine are highlighted as being high in patients in the AKI^(+)^ group compared to those in the AKI^(-)^ group, while fibrinogen values were decreased in AKI^(+)^. The results obtained after the analysis of the clinical urine tests demonstrate that the AKI^(+)^ group had higher values of leukocytes in the urine. It is also worth noting that there are high values of proteinuria in this group when compared to patients in the AKI^(-)^ group, although without statistical significance (Table 3).

### 3.2. Bothrops atrox Snakebite Patients Are Represented by a Sharp Inflammatory Profile, Regardless of Acute Kidney Injury Development

**Figure 2:**
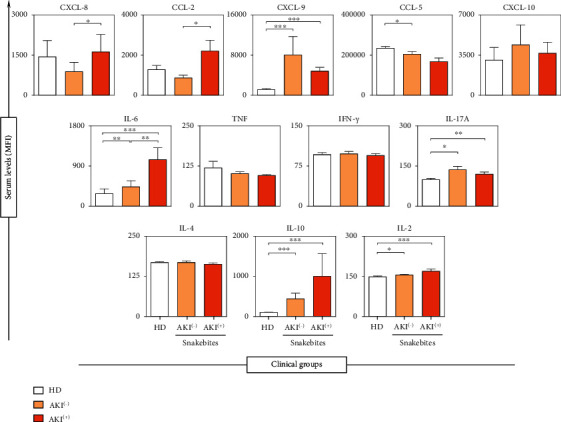
Concentration of soluble immunological molecules on admission of the patient (T0), in the groups AKI^(+)^, AKI^(-)^, and HD. ^∗^*p* < 0.01;  ^∗∗^*p* < 0.008;  ^∗∗∗^*p* < 0.0001. The results are expressed as the mean ± standard deviation in MFI (mean fluorescent intensity), and the statistical analysis was performed using the Kruskal-Wallis test followed by Dunn's test.

At T0, a significant increase in CXCL-9, IL-6, IL-2, IL-10, and IL-17A molecules was observed in the groups of patients who suffered *Bothrops* envenomation, regardless of AKI presence, when compared to the healthy donors (HD). In addition, a decrease in CCL-5 concentrations was observed in the group AKI^(-)^ compared to the HD group. When comparing the groups AKI^(-)^ and AKI^(+)^, CXCL-8, CCL-2, and IL-6 molecules showed higher levels in the AKI^(+)^group (Figure 2).

### 3.3. Ascendant Biomarker Signatures Evidenced a High Production of Immunological Molecules in Bothrops atrox Snakebite Patients, with Emphasis on the Selective Increase of CXCL-8 and CCL-2 in the AKI^(+)^ Group

**Figure 3:**
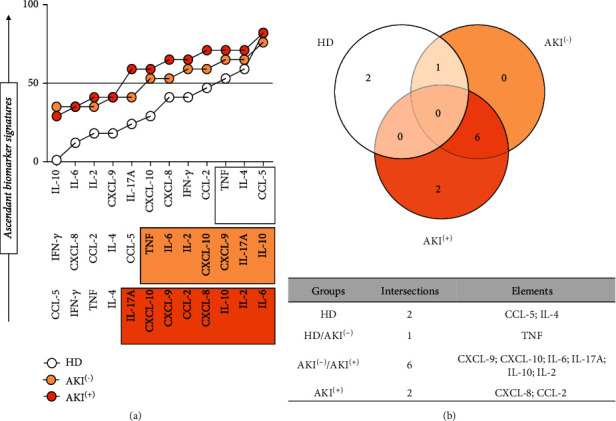
Ascendant signature of soluble immunological molecules presented by AKI(-) and AKI(+) patients. Performed based on the global median of each of the dosed cytokines and chemokines, using data from all patients (HD (healthy donors), AKI^(-)^ without acute kidney injury, and AKI^(+)^ with acute kidney injury). The global median of each molecule was used as a cut-off point, expressed in MFI (mean fluorescent intensity), thus classifying the individual as a “low” or “high” producer of the dosed molecules. Statistical significance was considered in all cases at *p* < 0.05. In the Venn diagram, it is possible to identify which molecules prove to be potential biomarkers because they are present in high concentrations only in the AKI^(+)^ group.

Additional analysis was carried out to identify potential biomarkers for AKI. For this purpose, we applied data mining approaches to generate immunological molecules signatures, where the original results, which were expressed as a continuous variable, were converted into categorical data to estimate the proportion of patients with high levels of soluble molecules, investigating their possible relationship with the development of AKI. Data mining was performed by selecting the set of biomarkers observed in more than 50% of individuals in each clinical group, as presented in Figure 3.

Based on this approach, a high production of IL-2, IL-6, IL-10, IL-17A, CXCL-8, CXCL-9, CXCL10, and CCL-2 molecules was observed in the patients of the AKI^(+)^ (Figure 3(a)). Furthermore, the analysis of the Venn diagram intersections showed that, at T0, there is a selective production of CXCL-8 and CCL-2 molecules in the group AKI^(+)^, thus highlighting their behavior as potential AKI biomarkers in these patients (Figure 3(b)).

### 3.4. Follow-Up of the Immunological Molecule Levels in B. atrox Snakebite Patients during the Hospitalization Period Demonstrated Distinct Behavior between AKI^(-)^ and AKI^(+)^ Groups

**Figure 4:**
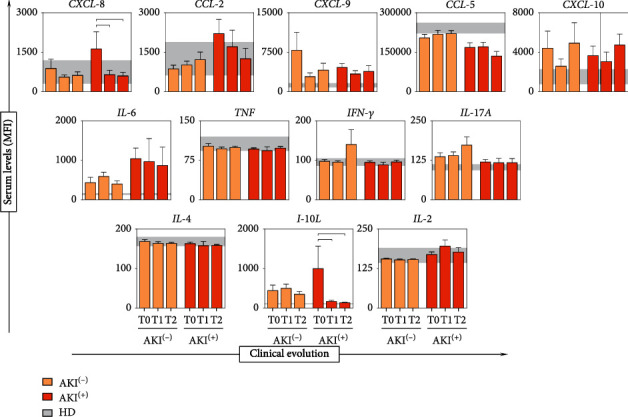
Analysis of the dynamics of soluble immunological molecule production during the clinical evolution of patients. In the background, the interquartile range [25–75] of the concentration of cytokines and chemokines in the HD healthy donors group is used as a baseline. The statistical difference between the groups AKI^(+)^ with acute kidney injury and AKI^(-)^ without acute kidney injury was considered when *p* < 0.05 (┌┐). The results are expressed as the mean ± standard deviation in MFI (mean fluorescent intensity), and statistical analysis was performed using the Kruskal-Wallis test followed by Dunn's posttest.

The dynamics of the production of soluble immunological molecules were analyzed on admission (T0) and 24 and 48 hours after antivenom administration (T1 and T2, respectively). The comparison between the times in each group demonstrates the behavior of chemokines and cytokines during the patients' hospital stay. A progressive increase in CCL-5, CCL-2, and IL-17A concentrations was observed in the AKI^(-)^ group, without statistically significant difference. CXCL-8, CCL-2, IL-6, and IL-10 constitutively decreased over time; however, only the CXCL-8 and IL-10 showed statistical significance in the group AKI^(+)^ (Figure 4).

### 3.5. Bothrops atrox Snakebite Patients Demonstrated Complex Biomarker Networks with Poor Interactions in the Circulating Serum of the AKI^(+)^ Group

**Figure 5:**
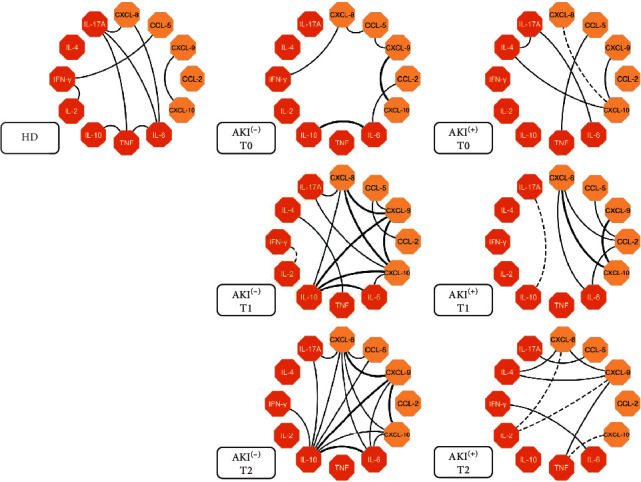
Network of soluble immunological molecules shows interactions occurring between groups in the follow-up of the study. Each color group is used to identify chemokines (yellow box) and cytokines (orange box). Dashed lines between molecules indicate a negative correlation while solid lines indicate a positive correlation. The thickness of these indicates the strength of the correlation. The correlation index (*r*) was used to categorize the strength of the correlation as either weak (*r* ≤ 0.35), moderate (*r* ≥ 0.36 to *r* ≤ 0.67) or strong (*r* ≥ 0.68).

Data analysis showed that patients who suffered snakebites (AKI^(-)^ and AKI^(+)^) have a network of interactions between the immunological molecules that are different to the profile displayed by the HD group (Figure 5). At T0, the AKI^(-)^ group showed a weak interaction between chemokines and cytokines. The inflammatory response started more clearly at T1 and T2, with moderate and strong correlations between the molecules, with emphasis on a chemotactic process during clinical evolution, in addition to a regulatory profile promoted by IL-10 at T2. The AKI^(+)^ patients showed a weak interaction between the molecules at T0, with an increase in interactions between chemokines and cytokines after application of antivenom (T1 and T2), highlighting the chemokines CXCL-8 and CCL-2 at T1, in addition to the absence of participation of the regulatory cytokine IL-10 during clinical follow-up.

## 4. Discussion

Kidney damage resulting from *Bothrops* envenomation is usually presented in the first hours after the bite [41, 42], which corroborates our findings since the AKI^(+)^ group developed this complication within the first 48 hours of hospitalization. Factors such as hypertension and diabetes increased LDH (lactate dehydrogenase), and bleeding at the bite site may be related to the development of AKI in *Bothrops* envenomation [34, 43], as shown in this study (Table 2). The AKI^(+)^ group showed high values of LDH compared to AKI^(-)^, along with an increase in creatinine levels, which is the main marker in renal function assessment [44, 45].

Hemostatic changes are the most common clinical signs after *Bothrops* envenomation and may be related to the development of AKI with the participation of procoagulant toxins and thrombin-like toxins [46, 47]. Castellanos et al., in a crosstalk study of the inflammation-coagulation axis, showed that 76.4% of patients who suffered *Bothrops* envenomation's had hypofibrinogenemia [48], thus reinforcing our findings (Table 2), since we observed a decrease in fibrinogen levels in the group AKI^(+)^ compared to AKI^(-)^. In addition, interactions between chemokines and cytokines in patients with hypofibrinogenemia are directly affected by fibrinogen levels, which connects the inflammatory response and coagulation induced by *Bothrops atrox* venom [48, 49]. Studies also suggest that thrombin and fibrin may act as important regulators of inflammation, and the nonregulation of their functions contributes to the development of thromboinflammatory events, which may play a role in AKI [50–52].

Microscopic analysis of urine is an important part of the assessment of patients with AKI, and it is used to differentiate a range of clinical conditions, aid in interventions, and improve the patient's clinical management [53, 54]. The high presence of leukocytes in the urine (pyuria) implies inflammation or an infectious process, with neutrophils being the most predominant [55]. In parallel, clinical conditions that increase protein levels in the glomerular filtrate or that decrease tubular reabsorption lead to increased protein in the urine (proteinuria) [56], which is usually seen after snakebites [57]. The presence of proteins such as hemoglobin, myoglobin, or immunoglobulins can lead to tubular necrosis due to obstruction of the proximal tubules, thus causing cell damage [23, 58, 59]. We observed that AKI^(+)^ patients (Table 3) showed high levels of leukocytes and proteins in the urine when compared to the AKI^(-)^ group, pointing to the existence of kidney damage.

Studies describe acute inflammation as a consequence of envenomation by *Bothrops* sp. [60, 61], and this may play a role in the initiation and extension of AKI [62], with the participation of immunological molecules in response to the action of toxins present in BaV [63]. *Bothrops* envenomation can induce an increase in the concentrations of molecules such as TNF-*α*, IL-6, IL-10, IFN-*γ*, and NO, and both PLA_2_ and MP can contribute to the production of cytokines, inducing alterations that are typical of inflammation [63, 64]. Ibiapina et al. demonstrated that in victims of envenomation by *Bothrops atrox* the levels of circulating inflammatory molecules are high before the antivenom administration, evidencing acute inflammation in response to the bite [37]. Our results (Figure 2) demonstrate an increase in the concentrations of CXCL-9, IL-6, IL-2, IL-10, and IL-17A in the groups that received bites from *B. atrox* (AKI^(+)^ and AKI^(-)^) when compared to the HD group, indicating the marked acute inflammatory response in these patients.

Renal vascular endothelial cells initiate early inflammatory responses in the injured kidney due to direct contact with toxins, and renal cells exposed to the venom mimic changes in the human body and, in response, produce mediators such as chemokines and cytokines that stimulate the migration of inflammatory cells to the kidneys [65, 66]. Experimental models demonstrate the direct effect of toxins that lead to morphological renal damage, and these are present in the glomerulus, proximal and distal tubules, and vascular tissue, among others [57]. In the first 24 hours after envenomation, neutrophils are mainly involved, followed by monocytes, with the involvement of endogenous mediators that lead to the activation of macrophages and regulation of the production of cytokines [67]. Ischemic renal failure is linked to increased neutrophil infiltration in the kidney, releasing substances that damage tubular cells [68]. Studies suggest that these cells mediate tubular damage in AKI, thus playing a key role in its development [69].

CXCL-8 exerts a chemoattractant effect for neutrophils and is related to proteinuria associated with kidney disease, and its urinary increase has been reported in acute phases of glomerulonephritis and nephropathies [70, 71]. In parallel, CCL2 has been described as an essential mediator in the process of renal injury. It appears in high concentrations in renal tissues of patients with glomerular diseases and interstitial nephritis while also being associated with macrophage/monocyte infiltration in renal pathogenesis [72, 73]. After a direct injury, resident dendritic cells, renal endothelial cells, tubular epithelial cells, podocytes, and others can produce CCL-2 and other mediators in response to damage [74, 75]. Increased CCL-2 in urine after *Bothrops erythromelas* envenomation was associated with renal inflammation and tubular damage/atrophy, mainly proximal and distal convoluted tubules [76].

Tissue damage after renal ischemia and reperfusion may be due, in part, to activation of inflammatory pathways and recruitment of leukocytes [77, 78]. Rouschop et al., demonstrated that the administration of anti-CD44 in mice reduced the influx of neutrophils into post-ischemic tissue and associated the reduction of these cells with the preservation of renal function [79]. The elevation of chemokines such as CXCL-8 may play an important role in recruiting neutrophils in the ischemic kidney, mediating this tissue damage by releasing cytokines, free radicals, and proteases [80]. We observed that the CXCL-8 molecule is elevated at T0 in the AKI^(+)^ group and progressively decreases during the hospitalization period of these patients (Figures 2 and 3). In addition, CXCL-8 was shown to be produced exclusively by this group, together with the CCL-2 molecule (Figure 4), which highlights these molecules as potential indicators of acute kidney injury resulting from *Bothrops* envenomation.

Rice et al. suggest that CCL-2 may be a possible biomarker for mononuclear inflammation after ischemia-induced AKI and showed that areas with increased CCL-2 are correlated with increased monocyte infiltration in the kidney [27]. IL-6 expression is correlated with the onset and severity of acute renal failure [28, 81, 82]. Furthermore, elevated levels of IL-6 and other cytokines were associated with the severity of *Bothrops* envenomation, thus leading to the development of moderate/severe symptoms as they increased [37, 83]. These data support our findings, as we observed that the CCL-2 molecule is increased at T0 in the AKI^(+)^ group, as it also occurs with CXCL-8 and IL-6 molecules (Figure 2), which are already described as elevated after *Bothrops* envenomation, thus influencing the increase in the acute inflammatory response in these patients.

IL-10 is characterized as a regulatory molecule that is capable of suppressing the activation of neutrophils and monocytes and consequently its production of chemokines, cytokines, and nitric oxide (NO), and it is also described as being related to the inhibition of inflammatory and cytotoxic pathways linked to acute kidney failure [84, 85]. IL-10 has been portrayed as being able to protect against ischemic AKI and against cisplatin-induced AKI by inhibiting the activation of genes that lead to inflammatory leukocyte activation and adhesion and NO production [86]. Our results show the progressive decrease of IL-10 in the AKI^(+)^ group, which may be related to the development of this complication in these patients. In addition, our data from the integrative analysis of immunological molecules demonstrated that the AKI^(+)^ group is characterized by the absence of interactions established by IL-10, a profile that is opposite to AKI^(-)^, and which exhibited a progressive increase in the number of interactions with other inflammatory molecules. This distinct behavior between AKI^(-)^ and AKI^(+)^ highlights the importance of IL-10 in controlling the immune response to venom toxins and reinforces its ability to protect against the development of AKI in these patients (Figure 5).

**Figure 6:**
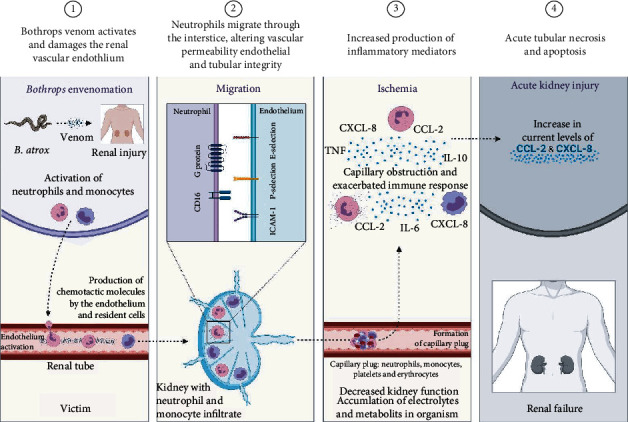
Probable pathogenic mechanism for the development of acute kidney injury after a *B. atrox* snakebite. Acute kidney injury initially results from the direct damage by the venom to the renal vascular endothelium. After activation of the endothelium, renal epithelial cells, tubular cells, and others produce chemotactic molecules, such as CCL-2 and CXCL-8, and synergistically increase the expression of p-selectins and cell adhesion molecule 1 (ICAM-1). Neutrophils and monocytes adhere to endothelial cells and migrate through the interstitium, causing changes in vascular permeability and compromising the cell integrity of the endothelium and renal tubules. A capillary plug is formed with the infiltration of neutrophils, monocytes, platelets, and red blood cells. Infiltrated neutrophils and monocytes release proinflammatory chemokines and cytokines, exacerbating the immune response, followed by alterations such as NTA and cell apoptosis that lead to decreased renal function with accumulation of metabolites and electrolytes in the body, resulting in acute renal failure in these patients. It is noteworthy that the cells represented in the figure were not evaluated in the present study.

A possible physiopathogenic model for AKI development in *Bothrops* envenomation is illustrated in Figure 6. Studies show that venom fractions may be responsible for the direct injury to kidney cells, with proteolytic enzymes inducing the destruction of renal structures through the generation of free radicals and activation and release of mediators such as chemokines, cytokines prostaglandins, and others [87]. Furthermore, direct nephrotoxicity was observed after inoculation of crude venom from *Bothrops leucurus*, *B. jararaca*, and *B. insularis*, which lead to cell death by necrosis and apoptosis of renal epithelial cells and proximal tubular cells, with acute tubular necrosis (ATN) being the most frequent [19, 41, 88, 89].

After activation of the renal vascular endothelium, it produces molecules such as CCL-2 and CXCL-8, in addition to increasing the expression of p-selectins and cell adhesion molecule 1 (ICAM-1), in which neutrophils and monocytes adhere to endothelial cells and migrate through the interstitium, causing changes in vascular permeability, compromising the cellular integrity of the endothelium and renal tubules [90–92]. The infiltrate of these cells leads to the formation of a capillary plug with the accumulation of neutrophils, monocytes, platelets, and red cells, in which neutrophils and monocytes release proinflammatory chemokines and cytokines, which promotes an exacerbated immune response that results in alterations such as ATN and cell apoptosis and culminates in acute kidney injury in these patients [75, 90, 93].

It is important to highlight that due to the insufficient volume of samples, this study has some limitations, such as the nondosing of molecules already described and which have a potential role in the development of AKI (IL-18, NGAL, and molecules of the complement system), as well as the lack of evaluation of the described cells (macrophages and neutrophils). However, the results presented here help understand this systemic complication, which is the main cause of death after *Bothrops* envenomation and has significant clinical importance.

## 5. Conclusion

Early identification of AKI is the main impetus for the search for new biomarkers since; in addition to facilitating a timelier intervention, early biomarkers can provide valuable information regarding likely mechanisms of this complex and heterogeneous disease. Cells such as neutrophils and monocytes play a central role in the body's defense; however, if not properly regulated, this same defensive role can lead to tissue damage and organ failure. Our results suggest that these cells are important in renal pathophysiology after *Bothrops* envenomation, together with their chemoattractant CCL-2 and CXCL-8, and demonstrate that they play a possible role as predictive biomarkers AKI in these patients.

## Figures and Tables

**Table 1:** Clinical and demographic characteristics of patients and individuals participating in the study.

Variable	HD *n* = 17	AKI^(-)^ *n* = 17	AKI^(+)^ *n* = 17
			
*Gender,n(%)*			
Male	16 (94)	14 (82)	15 (88)
Female	1 (6)	3 (18)	2 (12)
*Age (years, median (IQR))*	31 [23–63]	39 [24–57]	42 [22–39]
*Occurrence zone,n(%)*			
Rural	—	16 (94)	15 (88)
Urban	—	1 (6)	2 (12)
*Bite classification,n(%)*			
Mild	—	17 (100)	—
Severe	—	—	17 (100)
*Anatomical site of the bite,n(%)*			
Upper limbs	—	5 (29)	4 (23)
Lower limbs	—	12 (71)	13 (77)
*Previous snakebite,n(%)*			
Yes	—	3 (18)	—
No	—	14 (82)	17 (100)
*Adverse reaction to antivenom,n(%)*			
Yes	—	2 (12)	3 (18)
No	—	15 (88)	14 (82)
*Comorbidities,n(%)*			
Yes	—	1 (6)	4 (23)
No	—	16 (94)	13 (77)
*Time to medical assistance (hours,n(%))*			
0 – 3	—	12 (71)	10 (59)
4 – 6	—	5 (29)	2 (12)
>6	—	—	5 (29)
*Time to development of AKI (hours,n(%))*			
≤24	—	—	10 (59)
>24	—	—	7 (41)

**Table 2:** Laboratory characterization of the groups (AKI^(-)^) and (AKI^(+)^).

Laboratory variable	AKI^(-)^ *n* = 17	AKI^(+)^ *n* = 17	*p* value
Fibrinogen level (mg/dL, median (IQR))	207.6 (203.3-207.6)	82.6 (81.5-89.6)	0.0006
Platelets (×10^3^/mm^3^, median (IQR))	229.0 (191.5-276.0)	226.0 (181.5-256.0)	0.7961
Leukocytes (×10^3^/mm^3^, median (IQR))	8.75 (7.7-9.1)	9.90 (6.5-13.8)	0.5350
Erythrocytes (×10^6^/mm^3^, median (IQR))	4.82 (4.7-5.1)	4.70 (4.3-5.2)	0.3890
Hemoglobin (g/dL, median (IQR))	14.45 (13.9-15.4)	14.82 (12.9-15.7)	0.7304
Creatine kinase (IU/L, median (IQR))	176.0 (106.0-242.0)	208.0 (117.5-458.5)	0.1735
Lactate dehydrogenase (IU/L, median (IQR))	300.9 (200.1-300.2)	448.0 (346.0-707.0)	0.0005
Urea (mg/dL, median (IQR))	32.0 (25.5-36.0)	35.0 (26.0-59.0)	0.1050
Creatinine (mg/dL, median (IQR))	1.00 (0.90-1.25)	1.40 (1.15-1.80)	0.0036

The results are expressed as median (IQR), and the statistical analysis was performed using the Mann–Whitney test followed by the *T* test. Statistical significance was considered at *p* < 0.05 in all cases. Reference values: fibrinogen: 200-400 mg/dL; platelets: 130-400 × 10^3^/mm^3^; leukocytes: 4-10 × 10^3^/mm^3^; hemoglobin: 13.0-16.0 g/dL for males and 12.0-14.0 g/dL for females; creatine kinase: 24-190 IU/L; lactate dehydrogenase: 211-423 IU/L; urea: 10-45 mg/dL; creatinine: 0.5-1.2 mg/dL.

**Table 3:** Urine sediment analysis in groups (AKI^(-)^) and (AKI^(+)^).

Urine analysis	AKI^(-)^ *n* = 17	AKI^(+)^ *n* = 17	*p* value
*Density,n(%)*			
Normal	2 (12)	1 (6)	1.000
Abnormal	15 (88)	16 (94)	
*pH level,n(%)*			
Normal	15 (88)	14 (82)	1.000
Abnormal	2 (12)	3 (18)	
*Protein,n(%)*			
No	15 (88)	9 (53)	0.0570
Yes	2 (12)	8 (47)	
*Hemoglobin,n(%)*			
No	16 (94)	11 (64)	0.0854
Yes	1 (6)	6 (36)	
*Leukocytes,n(%)*			
No	14 (82)	6 (36)	0.0134
Yes	3 (18)	11 (64)	
*Crystals,n(%)*			
No	13 (76)	8 (47)	0.1571
Yes	4 (24)	9 (53)	
*Epithelial cells,n(%)*			
No	5 (30)	2 (12)	0.3983
Yes	12 (70)	15 (88)	

The results are expressed as total *n* (percentage), and the statistical analysis was performed using Fisher's exact test. Statistical significance was considered at *p* < 0.05 in all cases (reference values: pH: 5.5-7.0; density: 1025-1035). The other variables were classified only according to their presence (yes) or absence (no).

## Data Availability

The data used to support the findings of this study are included within the article.
